# *Tfam* Knockdown Results in Reduction of mtDNA Copy Number, OXPHOS Deficiency and Abnormalities in Zebrafish Embryos

**DOI:** 10.3389/fcell.2020.00381

**Published:** 2020-06-12

**Authors:** Auke B. C. Otten, Rick Kamps, Patrick Lindsey, Mike Gerards, Hélène Pendeville-Samain, Marc Muller, Florence H. J. van Tienen, Hubert J. M. Smeets

**Affiliations:** ^1^Department of Genetics and Cell Biology, Maastricht University, Maastricht, Netherlands; ^2^Department of Dermatology, University of California, San Diego, La Jolla, CA, United States; ^3^School for Oncology and Developmental Biology (GROW), Maastricht University Medical Centre, Maastricht, Netherlands; ^4^School for Mental Health and Neurosciences (MHeNS), Maastricht University Medical Centre, Maastricht, Netherlands; ^5^Maastricht Centre for Systems Biology (MaCSBio), Maastricht University Medical Centre, Maastricht, Netherlands; ^6^Laboratory of Organogenesis and Regeneration, Univérsité Liège, Liège, Belgium

**Keywords:** mtDNA bottleneck, zebrafish embryogenesis, OXPHOS deficiency, transcriptomics, TFAM, mitochondria

## Abstract

High mitochondrial DNA (mtDNA) copy numbers are essential for oogenesis and embryogenesis and correlate with fertility of oocytes and viability of embryos. To understand the pathology and mechanisms associated with low mtDNA copy numbers, we knocked down mitochondrial transcription factor A (*tfam*), a regulator of mtDNA replication, during early zebrafish development. Reduction of *tfam* using a splice-modifying morpholino (MO) resulted in a 42 ± 17% decrease in mtDNA copy number in embryos at 4 days post fertilization. Morphant embryos displayed abnormal development of the eye, brain, heart, and muscle, as well as a 50 ± 22% decrease in ATP production. Transcriptome analysis revealed a decrease in protein-encoding transcripts from the heavy strand of the mtDNA, and down-regulation of genes involved in haem production and the metabolism of metabolites, which appear to trigger increased rRNA and tRNA synthesis in the nucleoli. However, this stress or compensatory response appears to fall short as pathology emerges and expression of genes related to eye development are severely down-regulated. Taken together, this study highlights the importance of sufficient mtDNA copies for early zebrafish development. Zebrafish is an excellent model to manipulate the mtDNA bottleneck and study its effect on embryogenesis rapidly and in large numbers of offspring.

## Introduction

Mitochondria are responsible for producing the majority of cellular energy in the form of ATP. Depending on the tissue type and energy requirement, a cell can contain up to thousands of mitochondria, each having multiple copies of mitochondrial DNA (mtDNA). Of all cell-types, mtDNA copy numbers are the highest in oocytes, ranging between 100,000 and 400,000 copies in mammals, such as rodents, cows and humans, and up to 16.5 million copies in zebrafish (Otten and Smeets, 2015). The high mtDNA copy number in oocytes is established by an initial reduction during embryogenesis, called the mitochondrial bottleneck, followed by clonal expansion of the mtDNA during oogenesis appears to be important for successful fertilization and embryogenesis (Santos et al., 2006). In mice, the oocyte mtDNA copy number should be sufficient for normal development until implantation at day 4, and it has been demonstrated that oocytes with less than 50,000 mtDNA copies fail to resume development after implantation (Ebert et al., 1988; Wai et al., 2010). This negative correlation between mtDNA copy number and developmental competence of embryos has also been suggested for human oocytes (Yamamoto et al., 2010).

Mitochondrial transcription factor A (TFAM), a protein of the high mobility group box-family, is a key activator of mtDNA replication and transcription (Parisi et al., 1993) and is crucial for the regulation of mtDNA copy number (Larsson et al., 1998). A direct relation between the mtDNA copy number and the total TFAM protein level has been demonstrated in mice embryos with a heterozygous or homozygous disruption of the *Tfam* gene (Ekstrand et al., 2004). Homozygous TFAM knockout (KO) mouse embryos displayed a strong mtDNA depletion with severely reduced functioning of the electron transport chain (ETC) and died after gastrulation and implantation, while heterozygous KO TFAM mice had moderately reduced mtDNA levels and respiratory chain deficiency, which was strongest in the developing heart (Larsson et al., 1998). These studies demonstrated the importance of a sufficient mtDNA copy number during oogenesis and embryogenesis, but the mechanism by which a reduced mtDNA copy number affects embryogenesis is currently unknown.

Studying embryonic development in zebrafish overcomes ethical and practical obstacles associated with the use of human or mouse embryos. Zebrafish are vertebrates, >70% of human genes have at least one zebrafish orthologue, and the major tissues and organs are the same. Zebrafish are transparent during early development and have a high number of offspring. Organs develop within 5 days post fertilization and gene knockdown during early embryogenesis can be performed by injection of morpholino antisense oligonucleotides (MOs) (Pauli et al., 2015). Previously, we showed that the mitochondrial bottleneck during early development is highly conserved between zebrafish and mammals (Otten et al., 2016). In addition, the zebrafish *Tfam* protein is functionally homologous to its human counterpart and is expressed ubiquitously from the earliest stages of development (Artuso et al., 2012). In this study, we performed *tfam* knockdown during zebrafish embryogenesis in order to reduce the mtDNA copy number during early development. In this way, we created an animal model with a tuneable mtDNA bottleneck, which allows us to assess the effect of differences in mtDNA copy number on OXPHOS function and embryonic development and to determine the underlying mechanisms.

## Materials and Methods

### Zebrafish Maintenance and Procedures

Zebrafish (*Danio rerio*) were housed and raised in the zebrafish facility of the University of Liège as described before (Larbuisson et al., 2013). To retrieve eggs, wild-type adult male and female zebrafish were separated within the same breeding tank by a plastic divider the day before breeding. This separation was removed the next day after the light was turned on in order to allow natural mating and eggs were collected after spawning. Eggs were collected in Petri dishes containing 1× E3 medium for zebrafish at 28°C (580 mg/l NaCl, 27 mg/l KCl, 97 mg/l CaCl_2_⋅2H_2_O, 163 mg/l MgCl_2_⋅6H_2_O and 0.0001% methylene blue (Sigma-Aldrich), pH 7.2) (Larbuisson et al., 2013). Embryos were microscopically staged according to the embryonic development as described before (Kimmel et al., 1995). Unless stated otherwise, all reagents used in this study were obtained from Thermo Fisher Scientific.

### Tfam Knockdown Experiments

Antisense morpholino oligonucleotides (MO) were purchased from Gene Tools and micro-injected into one or two-cell stage embryos. A splice modifying MO was used, targeting the boundary of exon 2 and intron 2–3 of the zebrafish *tfam* gene (Ensemble: ENSDART00000092009, *tfam* splice-MO: 5′-CGGCAGATGGAAATTTACCAGGATT-3 ′). For controlling the effect of the MO injection, an equal concentration of a standard control-morpholino (Ctrl-MO: 5′-CCTCTTACCTCAGTTACAATTTATA-3′) was used in separate embryos of the same batch during each experiment. All MOs were dissolved in 1× Danieau buffer (58 mM NaCl, 0.7 mM KCl, 0.4 mM MgSO_4_, 0.6 mM Ca(NO_3_)_2_, and 5.0 mM HEPES pH 7.6) and 0.5% rhodamine dextran (Thermo Fisher) was added to the MO solution upon injection. At 1 hpf, 1 nl was injected into each embryo using microinjection (Bill et al., 2009). The next day, distribution of the rhodamine dextran dye was checked using fluorescence stereomicroscopy to verify proper injections. Only those embryos in which the rhodamine dextran dye was visible were used for follow-up investigations.

### Quantitative and Qualitative Analysis of Tfam RNA

Total RNA of 2–4 dpf zebrafish embryos was extracted using Trizol reagent and purified using the RNeasy Mini Kit (Qiagen), according to manufacturer’s instructions. cDNA synthesis was performed with 500 ng RNA in the presence of first strand buffer, 0.75 μg oligo-dT, 0.75 μg random hexamer-primer, 50 nmol dNTPs (GE Healthcare Life Sciences), 1 U RNAse inhibitor (RNAsin, Promega) and 5 U reverse transcriptase for 60′ at 42°C, followed by 5′ at 95°C. The effect on *tfam* splicing was assessed using RT-PCR amplification of 25 ng cDNA in a PCR mix contained under standard conditions, using 0.6 μM forward primer, 0.6 μM reverse primer (Supplementary Table S1). PCR conditions were 5′ denaturation at 94°C, 35 cycles of 1′ at 94°C, 1′ at 58°C and 1,5′ at 72°C, followed by 7′ at 72°C. The PCR product was sequenced by conventional Sanger sequencing. *tfam* gene expression was quantified by comparing the RNA expression ratio of *Tfam* RNA to *18S* RNA. Per reaction, 2.5 ng cDNA was amplified in a 10 μl reaction containing 1× Sensimix Sybr Hi-Rox reagents (Bioline) and 375 nM of both forward and reverse primer (Supplementary Table S1). Real-time PCR was performed on an ABI7900HT using the following settings: 10′ 95°C, 40 cycles of 15” 95°C and 1′ 60°C. The statistical analysis was performed by a Student’s *t*-test. *p*-values < 0.05 were considered significant.

### Quantification of mtDNA Copy Number

To determine mtDNA copy number, embryos were individually collected in sterile tubes and directly frozen without water. For DNA isolation, embryos were thawed and lysed for 4 h at 55°C in lysis buffer containing 75 mM NaCl, 50 mM EDTA, 20 mM HEPES, 0.4% SDS and 200 μg proteinase K (Sigma) while vortexing repeatedly. DNA precipitation was performed overnight at −20°C after adding 420 μl isopropanol. Following centrifugation, the DNA pellet was washed with 70% ethanol and dissolved in Tris-EDTA buffer. The relative mtDNA abundance was quantified by measuring the steady-state amount of mitochondrial ND1 and nuclear B2M. Per reaction, 20 ng DNA was used in a 10 μl reaction containing 1× Sensimix Sybr Hi-Rox reagents (Bioline) and 375 nM of both forward and reverse primer (Supplementary Table S1). Real-time quantification was performed on an ABI7900HT as described before. Statistical analysis was carried out using one-way analysis of variance (ANOVA) followed by the Bonferroni multiple comparisons test. *P*-values < 0.05 were considered significant.

### Imaging of Zebrafish Embryos

Zebrafish embryos were fixed at 4°C with paraformaldehyde for 4 h and standard paraffin serial sections of 4 μm thickness were prepared for immunostaining and hematoxylin-eosin (HE)-staining. Immunohistochemistry was performed following a microwave heat-induced antigen retrieval step for four times 5′ at 650 Watt (in Tris-EDTA, pH = 9.0) and was analyzed with the Dako REALTM EnVisionTM Detection System, Peroxidase/DAB, Rabbit/Mouse, using a Dako automated immunostaining instrument and protocols according to the manufacturer (Agilent, Santa Clara). Antibodies and conditions used are listed in Supplementary Table S1. HE-staining was performed to reveal the underlying embryonic structures. Paraffin slides were embedded in Entellan (Merck) and protected by cover slips (Knittel Glass). Microscopic images were taken by using the Nikon Eclipse E80 Imaging System (Nikon) at different magnifications. The orientation of fish was determined according to the Zebrafish Bio-Atlas^1^ (Cheng, 2004, 8 August).

### Oxygen Consumption Rate

At 4 dpf, chorion-free living fish embryos with a heartbeat and active swimming behavior upon touching were selected and collected individually. The oxygen consumption rate (OCR) (pmol/minute) was measured every minute for 15 min in a Seahorse XF24 system (Agilent/Seahorse Biosciences), according to the islet protocol of the manufacturer with 2 fish per well of a 24-well plate. Five minutes after starting the assay, 80 μM of Oligomycin (Sigma-Aldrich) was added and 60 μM for Antimycin & Rotenone (Sigma-Aldrich) in the injecting assay medium (1× E3 medium) was added 10 min after start. The OCR level at basal respiration (pmol/min) was calculated using the OCR values at the start respiration level (time-point 5 min) minus antimycin/rotenone (AMA) level at time-point 10 min. Maximal ATP production (pmol/min) was determined as start respiration level minus oligomycin (OMY) level at time-point 15 min. The statistical analysis was performed by a Student’s *t*-test. *p*-values < 0.05 were considered significant.

### Gene Expression and Pathway Analysis

For gene expression studies, *tfam* splice-MO and control-MO injected embryos and non-injected control embryos at 4 dpf, were individually collected in sterile tubes. Selection criteria were the same as for the OCR measurements. RNA from single embryos was isolated using the MagMax-96 for microarray kit (Ambion) and 200 ng RNA was labeled (Cyanine 3-CTP and Cyanine 5-CTP), fragmented and hybridized using the Low Input Quick Amp Labeling Kit, Two-colour (Ambion), according to manufacturer’s instructions. After labeling, amplified cRNA samples were purified using the RNeasy Mini kit (Qiagen) and Cyanine 3 and Cyanine 5 dye concentration, RNA absorbance ratio (260/280 nm) and cRNA concentration were quantified using the nanodrop 2000C (Thermo Fisher Scientific). Only samples with a yield > 0.825 μg and a specific dye activity >6.0 pmol/μg were used for fragmentation and hybridization. For fragmentation, 825 ng labeled cRNA was used and the final volume was adjusted to 41.8 μl with RNAse-free water, followed by the hybridization procedure, as described by the manufacturer (Ambion). Dye-swap hybridizations (2+2) were performed on microarray slides (4×44K zebrafish V3, Agilent) using gasket slides and a hybridization chamber and incubated for 17 h at 65°C and 10 rpm in the hybridization oven (Agilent Technologies). Slides were washed with Triton X-102, freshly added to the Wash Buffers. Microarray slides were scanned using a DNA Microarray scanner with *Surescan* High-Resolution Technology (Model 2565CA, Agilent). All microarray data is deposited in the GEO database, accession GSE146696.

The arrays contained 45,220 probes. Each probe identifier was transformed to Ensembl gene IDs (ENSDARG). This resulted in 36,156 probes containing a non-empty transcript ID of which 19,459 were unique transcripts and kept for the analysis. All transcripts were analyzed using a multivariate Gaussian linear regression MVN(μ, Σ) where μ is the mean, Σ is the covariance matrix $\left(\begin{array}{cccc} \sigma^{2}+\delta & \delta & \cdots & \delta \\ \delta & \ddots & \ddots & \vdots \\ \vdots & \ddots & \ddots & \delta \\ \delta & \cdots & \delta & \sigma^{2}+\delta \end{array}\right),\sigma^{2}$ is the variance, and δ is both the extra component of variance across subjects and the common covariance among responses on the same subject) including slide differences (Slide), dye swap (Dye), background level (Bg), injection (Inj), and a random effect. The inference criterion used for comparing the models is their ability to predict the observed data, i.e., models are compared directly through their minimized minus log-likelihood. When the numbers of parameters in models differ, they are penalized by adding the number of estimated parameters, a form of the Akaike information criterion (Akaike, 1973). For each transcript, a model containing the relevant covariates mentioned above (E(*y*) = Slide + Dye + Bg + Inj) was fitted in order to obtain a reference AIC. Then a model containing the treatment group (Trt) was fitted (E(*y*) = Slide + Dye + Bg + Inj + Inj:Trt). The transcript under consideration was found to be differentially expressed if the AIC of this second model decreased compared to the model not containing the treatment. These statistical analysis were performed using the freely available program R (Ihaka, 1996) and the publicly available libraries “rmutil” and “growth” (Lindsey, 1999). An unbiased Gene-ontology analysis and visualization of microarray data on biological pathways was performed using PANTHER (Protein Analysis THrough Evolutionary Relationships) with the *D. rerio* (Dr_Derby_Ensembl_80) gene product/protein database (Thomas et al., 2003; Mi et al., 2017). Differentially expressed genes (DEGs) were mapped to unique Entrez Gene IDs and to Gene Ontology (GO) classes, using the PANTHER Overrepresentation Test (Released 2018-02-03) enrichment method of PANTHER version 13.1.

## Results

### Tfam Knockdown Results in Decreased mtDNA Content and Mitochondrial ATP Production

**FIGURE 1:**
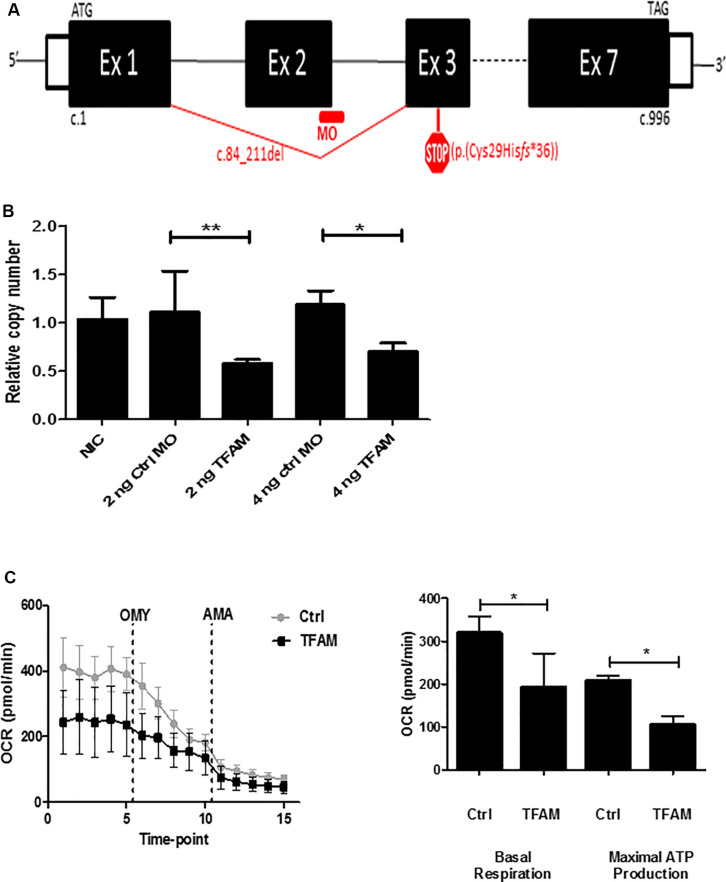
Knockdown of TFAM in zebrafish embryos. Zebrafish embryos injected with indicated amount of either Control-morpholino (Ctrl-MO) or *tfam* splice-morpholino *tfam* splice-MO at 1 hpf and analyzed at 4 dpf. **(A)** *tfam* splice-morpholino (MO) at the 3′ splice site of exon 2 causes deletion of exon 2, which predicts a frame-shift and premature stop codon (p.(Cys29Hisfs*36)) (figure is not on scale). **(B)** The relative copy number assessed by mitochondrial ND1/nuclear B2M ratio. Data are normalized to the NIC embryos (*n* = 7). Bars indicate mean values with SD, *n* = 20 per 2 ng injected condition and *n* = 16 per 4 ng injected condition. *P-*values are calculated using ANOVA followed by Bonferroni’s Multiple Comparison Test to assess copy number, *P-value < 0.05, **P-value < 0.01. **(C)** The oxygen consumption rate (OCR) measured by Seahorse XF24 in order to assess basal respiration and maximal ATP production capacity at 4 dpf using 2 ng MO. The statistical analysis was performed by using a student *t*-test (*P* < 0.05), *n* = 10 per *tfam* splice-MO condition and *n* = 9 per Ctrl-MO condition.

To establish decreased mtDNA copy number, we microinjected an antisense splice-MO targeting *tfam*-mRNA in zebrafish embryos. Using RT-PCR and Sanger sequencing of cDNA from *tfam* splice-MO-injected 4 dpf embryos (*n* = 6/group), we showed that the *tfam* splice-MO causes a 128 base-pair deletion of exon 2 (c.84_211del), predicted to cause a frameshift and a premature stop codon (p.(Cys29Hisfs^∗^36)) (Figure 1A and Supplementary Figure S1). Quantitative PCR analysis of *tfam* exon 5 and exon 6–7 showed, respectively, 59 ± 2% and 60 ± 5% decrease in expression in *tfam* splice-MO injected embryos at day 4 (mean ± SD, *n* = 6/group). qPCR analysis of *tfam* exon 2 expression showed a reduction of 80 ± 3% at day 4 in zebrafish (*n* = 6/group) injected with 2 ng *tfam* splice-MO compared to the Ctrl-MO (Supplementary Figure S2). This indicates that ∼60% of *tfam* RNA is subjected to nonsense-mediated decay and that of the residual ∼40% *tfam* RNA, only half is exon 2-containing wild-type RNA (only 20% of normal *tfam* amount). Next, we assessed the mtDNA copy number in non-injected, Ctrl-MO injected and *tfam* splice-MO injected embryos (*n* = 20 in each 2 ng group, and *n* = 16 in each 4 ng group). At 4 dpf, a significant, 42 ± 17% (mean ± SD) decrease in the mtDNA copy number was observed in embryos injected with either 2 ng or 4 ng *tfam* splice-MO (Figure 1B). In contrast, no significant differences in mtDNA copy number were apparent between groups at 2 dpf (Supplementary Figure S3). To assess if decreased mtDNA copy number also affected mitochondrial capacity, we measured the OCR at 4 dpf. As shown in Figure 1C, both the OCR of the basal respiration and the ATP production were significantly decreased in the 2 ng *tfam* splice-MO treated zebrafish (*n* = 10) compared with the 2 ng control-MO treated group (*n* = 9) (*p* < 0.05) by respectively, 40 ± 25 and 50 ± 22% (mean ± SD).

### Decreased mtDNA Copy Number Causes Brain, Eye, Heart, and Muscle Abnormalities

At 4 dpf, morphant embryos displayed morphological abnormalities compared to control embryos. The macroscopic phenotype included overall oedema, curved tails, necrotized yolk sacs and small eyes (Supplementary Figure S4). Fish injected with 4 ng *tfam* splice-MO were more severely affected than 2 ng *tfam* splice-MO (*n* = 100 injected per condition), as they had a higher count for oedema, curved tails, necrotized yolk sac, small eyes, and developmental delay. The percentage of dead embryos was <1% when injecting 2 or 4 ng of *tfam MO-*injections at 4 dpf (Supplementary Figure S4). In contrast, injection of 6 ng of *tfam* splice-MO showed >70% of embryonic lethality due to severe developmental complications. Macroscopic inspection of *tfam* splice-MO injected embryos showed that all observed pathologies manifested at 3 or 4 dpf, whereas development was apparently normal at 2 dpf. These morphological changes parallel the decrease in mtDNA copy number that is observed at 4 dpf, but not at 2 dpf (Figure 1B and Supplementary Figure S3). Since the decrease in mtDNA content was comparable with 4 ng *tfam* splice-MO, the dosage of 2 ng *tfam* splice-MO was used in all following experiments, reducing the risk of non-specific observations.

**FIGURE 2:**
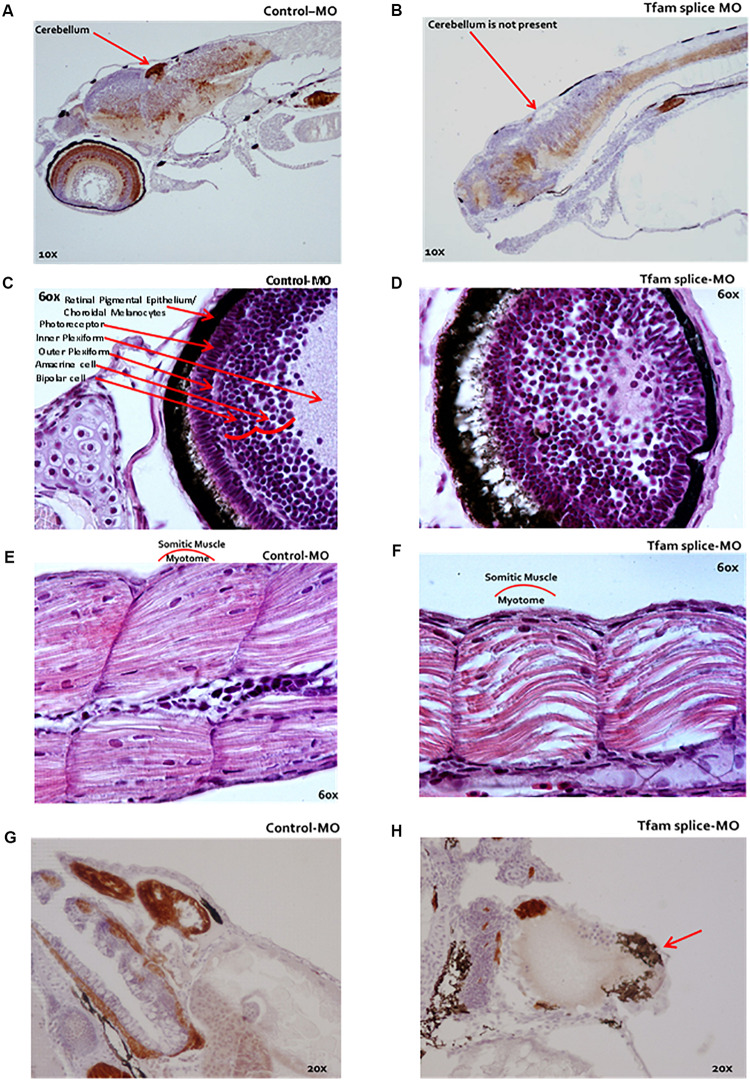
Microscopy of zebrafish embryos following TFAM knockdown. **(A–H)** Microscopic analysis following serial HE-staining of zebrafish embryos at 4 dpf, which were injected at 1 hpf with either 2 ng *tfam* splice-MO **(B–E)** or 2 ng Ctrl-MO, **(A,C,E,G)** *n* = 13 each condition. **(B)** The cerebellum was missing in the phenotype or a delayed development of this cerebellum in these fish**. (D)** The eyes were smaller and the eye layers were less developed. **(F)** A muscle phenotype was clearly visible in the dorsal fin, especially in the myotomic area, which was less compact in these somatic muscles. **(H)** The fish displayed a marked pericardial oedema (red arrow). They did not develop a normally looped heart as in Ctrl-MO **(G)**, but the hearts were dilated in the phenotypes **(H)**.

Microscopic evaluation of zebrafish (*n* = 13 per condition) with reduced mtDNA copy number using 2 ng *tfam* splice-MO showed impaired development compared to control embryos (Figure 2). Brain size was decreased with no or less well-developed cerebellum (Figure 2B), and the eyes were smaller and the different layers were less-well organized compared to controls (Figure 2D). Also, the organization of the myotomic area was less compact with the skeletal muscle fibres being thinner and disorganized (Figure 2F). Finally, morphant fish displayed marked pericardial oedema, alongside with a dilated non-looped heart (Figure 2H).

### Genes and Pathways Altered by Tfam Knockdown

To characterize the underlying pathogenic processes due to *tfam* knockdown and subsequent decrease in mtDNA copy number, we compared global gene expression profiles at 4 dpf of zebrafish injected with 2 ng of either *tfam* splice-MO (*n* = 12) or Ctrl-MO (*n* = 12). A group of non-injected control zebrafish was also included (*n* = 9) to control for the injection effect in the microarray analysis and allow identification of transcripts altered in the *tfam* splice-MO zebrafish compared with the Ctrl-MO zebrafish. A total of 19,459 transcripts were present on the array (Supplementary Table S2), of which 16,631 (85.5%) had a signal intensity higher than twice the background signal in both conditions or a signal that was at least three times higher than the background signal in either the group of *tfam* splice-MO zebrafish or the group of Ctrl-MO zebrafish. Of these, 3,158 transcripts (19.3%) were differentially expressed between the two groups with a fold change (FC) >50%. Expression of 2,063 transcripts (64.3%) was increased (FC ≥ 1.50), while expression of 1,145 transcripts (35.7%) was decreased in the *tfam* splice-MO injected embryos (FC ≤ 0.67). In order to identify significantly altered pathways by using gene ontology analysis, Protein ANalysis THrough Evolutionary Relationships (PANTHER) was used. The microarray contained 19,459 transcripts, of which 17,395 could be annotated (89.4%) and were included in the PANTHER analysis. A total of 18 significantly enriched GO biological processes (FDR < 0.05) were identified and these were manually grouped into five groups including the underlying GO terms, namely: tRNA processing, ribosome biogenesis, RNA surveillance and catabolism, drug metabolism, and visual perception (Table 1 and Supplementary Table S4). As the RNA-related processes differ for nuclear and mtDNA genes and for that reason should not be considered as one group, we also listed the mitochondria-related processes, characterized by mitochondria-related GO terms (Table 2 and Supplementary Table S5). Most of the mitochondria related GO terms were small, comprising less than 50 measured genes, but sufficient number of genes were interrogated to conclude that these processes were not significantly enriched by the mtDNA reduction. However, as expression of mtDNA encoded proteins, which is regulated by TFAM, was not covered in a GO term, we checked the expression of the 8 mtDNA protein encoding genes manually. The 8 mtDNA proteins encoding transcripts of the H-strand were significantly decreased, whereas the single L-strand transcript was unaltered (Supplementary Table S3). The remainder four protein encoding transcripts of the H-strand were not present on the array (atp6, atp8, cox 3, and cytb).

**TABLE 1:** Significantly altered GO biological process terms.

Description	Measured (*n*)	Changed (*n*)	Fold Enrichment	FDR
**tRNA processing**				
GO:0006396 RNA processing	426	106 (Cm = 15, Cn = 91)	1.52	2.4E-02
→ GO:0034660 ncRNA metabolic process	260	88 (Cm = 30, Cn = 58)	2.06	5.2E-06
→ GO:0006399tRNA metabolic process	118	44 (Cm = 22, Cn = 22)	2.27	1.4E-03
→ GO:0009451RNA modification	88	32 (Cm = 9, Cn = 23)	2.21	2.6E-04
→ GO:0034470 ncRNA processing	197	72 (Cm = 14, Cn = 58)	2.22	4.3E-06
→ GO:0008033tRNA processing	73	27 (Cm = 7, Cn = 20)	2.25	3.8E-02
**Ribosome biogenesis**				
GO:0022613 Ribonucleoprotein complex biogenesis	239	69 (Cm = 14, Cn = 55)	1.76	1.0E-02
→ GO:0042254 Ribosome biogenesis	162	60 (Cm = 14, Cn = 46)	2.25	4.3E-05
→ GO:0006364 rRNA processing	105	43 (Cm = 6, Cn = 37)	2.49	2.0E-04
**RNA surveillance and catabolism**				
GO:0071025 RNA surveillance	9	9 (Cm = 0, Cn = 9)	6.09	2.3E-02
→ GO:0071027 Nuclear RNA surveillance	8	8 (Cm = 0, Cn = 8)	6.09	3.4E-02
→ GO:0071028 Nuclear mRNA surveillance	8	8 (Cm = 0, Cn = 8)	6.09	3.6E-02
→ GO:0034475 U4 snRNA 3′-end processing	8	8 (Cm = 0, Cn = 8)	6.09	3.2E-02
→ GO:0016075 rRNA catabolic process	11	9 (Cm = 0, Cn = 9)	4.98	3.6E-02
**Drug metabolism**				
GO:0042737 Drug catabolic process	85	33 (Cm = 2, Cn = 31)	2.36	1.1E-02
**Visual perception**				
GO:0007601 Visual perception	83	31 (Cm = 2, Cn = 29)	2.27	2.3E-02
→ GO:0050953 Sensory perception of light stimulus	88	31 (Cm = 2, Cn = 29)	2.14	3.5E-02

*GO terms biological process with FDR < 0.05 were considered to be significantly changed. Cm are changed genes that are linked to mitochondria; Cn are changed genes linked to the nucleus; FDR is false discovery rate.*

**TABLE 2:** Mitochondria-related GO biological processes.

Description	Measured (*n*)	Changed (*n*)	Fold enrichment
GO:0007005 mitochondrion organization	152	15	0.60
GO:0007007 inner mitochondrial membrane organization	20	2	0.61
GO:0007008 outer mitochondrial membrane organization	1	0	
GO:0006390 mitochondrial transcription	8	2	1.52
GO:0032543 mitochondrial translation	27	8	1.80
GO:0006626 protein targeting to mitochondrion	32	1	0.19
GO:0070585 protein localization to mitochondrion	32	1	0.19
GO:0042775 mitochondrial ATP synthesis coupled electron transport	41	7	1.04
GO:0006119 oxidative phosphorylation	46	9	1.19
GO:0033108 mitochondrial respiratory chain complex assembly	25	2	0.49
GO:0015986 ATP synthesis coupled proton transport	20	1	0.30
GO:0006979 response to oxidative stress	70	9	0.78
GO:0006839 mitochondrial transport	73	6	0.50
GO:1990542 mitochondrial transmembrane transport	44	3	0.41
GO:0008053 mitochondrial fusion	12	3	1.52
GO:0000266 mitochondrial fission	16	1	0.38
GO:0000422 autophagy of mitochondrion	27	2	0.45
GO:0000002 mitochondrial genome maintenance	6	1	1.01
GO:0009117 nucleotide metabolic process (DNA replication)	282	46	0.99

*The false discovery rate (FDR) was > 0.0.5 for all processes.*

## Discussion

A high mtDNA copy number is critical for normal embryonic development. A key regulator of the mtDNA copy number is TFAM, as TFAM initiates mtDNA replication by its capability to wrap, bend and unwind the mtDNA (Ekstrand et al., 2004), a function conserved in zebrafish (Howe et al., 2013). In order to study the effect of a decreased mtDNA copy number during zebrafish embryonic development, we performed *tfam* knock-down using a splice-morpholino (MO). We demonstrated that the *tfam* splice-MO induced skipping of exon 2, leading to a frameshift and a premature stop codon (p.(Cys29Hisfs^∗^36)) (Figure 1A) and to nonsense mediated decay. RNA expression of normal *tfam* is reduced to 20% of control values. At 3 and 4 dpf, morphant zebrafish have a decreased mtDNA copy number and are less well developed. This correlates with the previous observations that in wild-type zebrafish larvae an increase in mtDNA copy number only becomes apparent between 2 and 3 dpf (Otten et al., 2016) indicating that, in the absence of mtDNA replication, mtDNA copy number and OXPHOS capacity becomes critically low at 3–4 dpf, as an increase is required for embryonic tissue differentiation and organogenesis. This is a critical phase of development, when there is a high energy demand and a metabolic switch from glycolysis to OXPHOS takes place (Hance et al., 2005; Stackley et al., 2011). This necessity is illustrated by the aberrant eye and muscle development, pericardial oedema and reduced brain size that were observed in morphant zebrafish (Figure 2). The heart and the brain are the first organs to develop (Glickman and Yelon, 2002) and therefore the first to be affected by OXPHOS defects. Moreover, as in a normal heart >30% of the cardiac muscle volume is occupied by mitochondria, the deficit of mtDNA (and subsequently mitochondria) also hampers proper alignment of the muscle fibers in the developing heart, creating structural problems as well (Ventura-Clapier et al., 2011). In homozygous *Tfam* KO mice, OXPHOS was abolished due to a severe mtDNA depletion, causing a complete failure of organogenesis and embryonic death (Larsson et al., 1998). Heterozygous KO mice were viable, but showed an OXPHOS deficiency in the developing heart and a dilated cardiomyopathy and conduction block later in life (Larsson et al., 1998; Powell et al., 2015). The severity of our model seems to fit somewhere in between, as wild-type *tfam* levels are around 20% of control level at the day of analysis. Noticeably, MOs establish only a transient knockdown, which is not the case for a homozygous or heterozygous knockout of the gene, prohibiting a proper comparison later in life.

**FIGURE 3:**
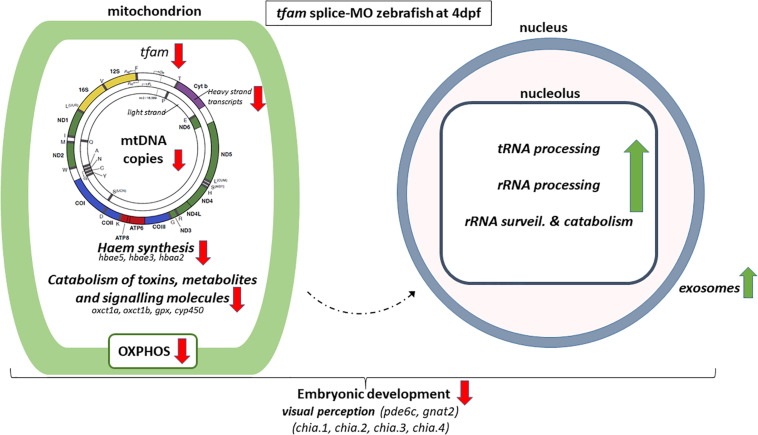
Schematic overview of alterations in *tfam* splice-MO zebrafish at 4dpf. The arrow indicates if a process is upregulated (green) or down-regulated (red). Altered gene expression processes and genes are shown in italic. The mitochondrial DNA figure is adapted from Amorim et al. (2019).

In order to unravel the biological processes that were altered, we performed global gene expression profiling and subsequent gene ontology (GO) analysis (Table 1 and Figure 3). Despite the observed reduction in OXPHOS function, the gene ontology term comprising mitochondrial respiratory chain components was not significantly changed, neither were critical mitochondrial processes (Table 2). However, gene expression was decreased of 8 out of the 9 mtDNA protein encoding transcripts, all encoded by the H-strand, whereas the one transcript on the L-strand was unaltered. This most likely this reflects a switch or preferable transcription from the Light-Strand Promoter (LSP) that is induced by low amounts of *tfam* and which also primes H-strand replication (Shutt et al., 2011), but which in our case fails to increase mtDNA copy number due to the persisting lack of *tfam*.

The GO term drug catabolism was significantly changed, comprising several downregulated genes linked to mitochondrial function, including embryonic hemoglobin production. As haem is produced by mitochondria (Wagener et al., 2003), this could be explained by the decreased amount of mtDNA and subsequently mitochondria at the onset of definitive haematopoiesis, initiating between 1 to 2 dpf (Murayama et al., 2006; Bertrand et al., 2007; Zhang and Rodaway, 2007; Zhang and Hamza, 2018). Obviously, deficiencies in haematopoiesis during development might have downstream effects as well. Other mitochondria-related genes of this GO term were decreased, such as *oxct1a*, *oxct1b*, members of *cyp450* family 2 and *gpx1a*, which are related to the metabolism of fatty acids and ketone bodies, exogenous drug catabolism and hydrogen peroxide degradation in the mitochondria. The decrease in mtDNA copy number and subsequent mitochondrial dysfunction appears to result in enrichment of GO terms related to rRNA synthesis, ncRNA processing, and ribosomal assembly (Table 1), and the vast majority of genes in these GO terms were upregulated (Supplementary Table S4). In addition, the GO term tRNA processing was also significantly enriched. In general, expression of genes belonging to these GO terms is increased, including a number of genes that are linked to mitochondrial function, like *mto1* (1.64), *trmt10c* (1.55), and *elac2* (1.61) (Haack et al., 2013; Metodiev et al., 2016; Fakruddin et al., 2018). Increased tRNA synthesis was also observed by Torrent et al. (2018) upon applying diverse stress conditions to *Saccharomyces cerevisiae*, demonstrating the regulation of tRNA abundance upon cellular stress. However, future research is needed to identify if the observed activation nucleolar processes is caused by mitochondrial stress or is a reaction to compensate for the reduction in mitochondrial transcripts. The resulting disbalance between mitochondrial transcripts and proteins, and nuclear encoded proteins that migrate into the mitochondria might be a factors as well. Also, gene expression of 8 out of 9 exosome components, belonging to GO term ribosomal biogenesis, were significantly upregulated in *tfam* splice-MO zebrafish. Like increased rRNA and tRNA expression, upregulation of the exosome components seems to be a compensatory or stress-induced mechanism, as exosomes contain and shuttle mRNA, and are important in intercellular communication and developmental patterning (Wan et al., 2012; Boczonadi et al., 2014; McGough and Vincent, 2016). Future assessment of the zebrafish exosome composition is needed to provide further insight into this novel mechanism by which decreased mtDNA copy number affects exosome signalling and composition.

Since clear morphological abnormalities were apparent in *tfam* splice-MO zebrafish at 4 dpf (Figure 2), the nucleolar activation upon mitochondrial deficiency was obviously insufficient to compensate. At gene expression level in *tfam* splice-MO zebrafish at 4 dpf, GO terms related to vision and sensitivity to light were significantly changed. Retinal development is critical between 32 and 74 hpf and normally largely completed at 4 dpf (Richardson et al., 2017). This window coincides with the decrease in mtDNA copy number in *tfam* splice-MO zebrafish, explaining the down-regulation observed. These extremely down-regulated eye genes are mainly involved in processes related to response to light stimulus (Supplementary Table S4), like *pde6c* (0.05) and *gnat2* (0.09). Zebrafish mutants of these genes respectively cause retinal and retinal pigment epithelial disease and cone-rod dystrophy (Brockerhoff et al., 2003; Richardson et al., 2017). Additional down-regulated opsins genes related to the photoreceptor function are listed in our zebrafish data (Supplementary Table S4). The lack of significant changes in pathways linked to cardiac, muscle or brain development, suggests that although pathology clearly manifested, we are still observing the processes preceding the pathology and not those resulting from it in the pathway analysis. In addition to defective eye development, a number of temporally expressed developmental genes are downregulated in *tfam* splice-MO zebrafish at 4 dpf, such as chitinase (Chia) 1, 2, 3 and 4. Expression of *chia.1*, *chia.2* and *chia.3*, which belong to the GO term drug metabolism, normally drastically increases at 4 and 5 dpf, while chia.4 is more stably expressed. Chitinases are suggested to play a role in early embryo immunity and chitinase inhibition has been shown to cause development disruption in zebrafish, characterized by hampered trunk and tail formation (Semino and Allende, 2000).

In conclusion, inhibition of tfam expression during early zebrafish development leads to a decrease in mtDNA copy number, OXPHOS capacity, and mtDNA encoded transcripts and haem production, affecting the oxygen supply in the developing embryo. This triggers a compensation mechanism or stress response in the nucleoli at the level of rRNA and tRNA biogenesis and translation initiation, but this compensation mechanism falls short, as pathology emerges (Figure 3). The results of this study in zebrafish are comparable to other animal models, thereby validating our model, but they also take it a step further due to the clear advantages of the zebrafish model, being a high number of offspring, a rapid development and an optical clarity during development combined with easy genetic interventions. Being able to tune the mtDNA content, we can now manipulate the mtDNA bottleneck and study in sufficient numbers the effects on processes, like organogenesis, fertility and mtDNA mutation rate.

## Data Availability Statement

All microarray data is deposited in the GEO database, accession GSE146696.

## Ethics Statement

The animal study was reviewed and approved by the Committee of Animal Research of the University of Liège.

## Author Contributions

FT and HS designed and supervised the project. AO and RK designed, performed most of the experiments, and coordinated collaborations with PL, MG, HP-S, and MM. PL designed, assigned, and supplied the transcriptomic computational analysis. RK, FT, and HS determined PANTHER pathway gene-expression analysis on transcriptomic data. AO and RK performed the Tfam knockdown experiments as quantitative and qualitative analysis, imaging embryos, oxygen consumption rate measurements. MG assisted on the design and scientific input on the manuscript. HP-S and MM assisted on the design and scientific input in the manuscript and were coordinating the housing and injections of the zebrafish embryos. All authors contributed to the ideas to the project.

## Conflict of Interest

The authors declare that the research was conducted in the absence of any commercial or financial relationships that could be construed as a potential conflict of interest.

## Footnotes

1 http://bio-atlas.psu.edu

## References

[B1] Akaike H. (1973). “Information theory and an extension of the maximum likelihood principle,” in *Proceedings of the Second International Symposium on Inference Theory*, eds Petrov B. N. Csàki F. (Budapest: Akadémiai Kiadó), 267–281.

[B2] Amorim A. Fernandes T. Taveira N. (2019). Mitochondrial DNA in human identification: a review. *PeerJ.* 7:e7314. 10.7717/peerj.7314 31428537 PMC6697116

[B3] Artuso L. Romano A. Verri T. Domenichini A. Argenton F. Santorelli F. M. (2012). Mitochondrial DNA metabolism in early development of zebrafish (*Danio rerio*). *Biochim. Biophys. Acta* 1817 1002–1011. 10.1016/j.bbabio.2012.03.019 22465854

[B4] Bertrand J. Y. Kim A. D. Violette E. P. Stachura D. L. Cisson J. L. Traver D. (2007). Definitive hematopoiesis initiates through a committed erythromyeloid progenitor in the zebrafish embryo. *Development* 134 4147–4156. 10.1242/dev.012385 17959717 PMC2735398

[B5] Bill B. R. Petzold A. M. Clark K. J. Schimmenti L. A. Ekker S. C. (2009). A primer for morpholino use in zebrafish. *Zebrafish* 6 69–77. 10.1089/zeb.2008.0555 19374550 PMC2776066

[B6] Boczonadi V. Muller J. S. Pyle A. Munkley J. Dor T. Quartararo J. (2014). EXOSC8 mutations alter mRNA metabolism and cause hypomyelination with spinal muscular atrophy and cerebellar hypoplasia. *Nat. Commun.* 5:4287. 10.1038/ncomms5287 24989451 PMC4102769

[B7] Brockerhoff S. E. Rieke F. Matthews H. R. Taylor M. R. Kennedy B. Ankoudinova I. (2003). Light stimulates a transducin-independent increase of cytoplasmic Ca2+ and suppression of current in cones from the zebrafish mutant nof. *J. Neurosci.* 23 470–480. 10.1523/jneurosci.23-02-00470.2003 12533607 PMC6741873

[B8] Cheng K. C. (2004). A life-span atlas for the zebrafish. *Zebrafish* 1:69. 10.1089/zeb.2004.1.69 18248218

[B9] Ebert K. M. Liem H. Hecht N. B. (1988). Mitochondrial DNA in the mouse preimplantation embryo. *J. Reprod. Fertil.* 82 145–149. 10.1530/jrf.0.0820145 3339575

[B10] Ekstrand M. I. Falkenberg M. Rantanen A. Park C. B. Gaspari M. Hultenby K. (2004). Mitochondrial transcription factor A regulates mtDNA copy number in mammals. *Hum. Mol. Genet.* 13 935–944. 10.1093/hmg/ddh109 15016765

[B11] Fakruddin M. Wei F. Y. Suzuki T. Asano K. Kaieda T. Omori A. (2018). Defective mitochondrial tRNA taurine modification activates global proteostress and leads to mitochondrial disease. *Cell Rep.* 22 482–496. 10.1016/j.celrep.2017.12.051 29320742

[B12] Glickman N. S. Yelon D. (2002). Cardiac development in zebrafish: coordination of form and function. *Semin. Cell. Dev. Biol.* 13 507–513. 10.1016/s1084952102001040 12468254

[B13] Haack T. B. Kopajtich R. Freisinger P. Wieland T. Rorbach J. Nicholls T. J. (2013). ELAC2 mutations cause a mitochondrial RNA processing defect associated with hypertrophic cardiomyopathy. *Am. J. Hum. Genet.* 93 211–223. 10.1016/j.ajhg.2013.06.006 23849775 PMC3738821

[B14] Hance N. Ekstrand M. I. Trifunovic A. (2005). Mitochondrial DNA polymerase gamma is essential for mammalian embryogenesis. *Hum. Mol. Genet.* 14 1775–1783. 10.1093/hmg/ddi184 15888483

[B15] Howe K. Clark M. D. Torroja C. F. Torrance J. Berthelot C. Muffato M. (2013). The zebrafish reference genome sequence and its relationship to the human genome. *Nature* 496 498–503. 10.1038/nature12111 23594743 PMC3703927

[B16] Ihaka R. G. R. (1996). R: A language for data analysis and graphics. *J. Comput. Graph. Stat.* 5 299–314.

[B17] Kimmel C. B. Ballard W. W. Kimmel S. R. Ullmann B. Schilling T. F. (1995). Stages of embryonic development of the zebrafish. *Dev. Dyn.* 203 253–310. 10.1002/aja.1002030302 8589427

[B18] Larbuisson A. Dalcq J. Martial J. A. Muller M. (2013). Fgf receptors Fgfr1a and Fgfr2 control the function of pharyngeal endoderm in late cranial cartilage development. *Differentiation* 86 192–206. 10.1016/j.diff.2013.07.006 24176552

[B19] Larsson N. G. Wang J. Wilhelmsson H. Oldfors A. Rustin P. Lewandoski M. (1998). Mitochondrial transcription factor A is necessary for mtDNA maintenance and embryogenesis in mice. *Nat. Genet.* 18 231–236. 10.1038/ng0398-231 9500544

[B20] Lindsey J. (1999). *Models for Repeated Measurements*, 2nd Edn Oxford: Oxford University Press.

[B21] McGough I. J. Vincent J. P. (2016). Exosomes in developmental signalling. *Development* 143 2482–2493. 10.1242/dev.126516 27436038

[B22] Metodiev M. D. Thompson K. Alston C. L. Morris A. A. He L. Assouline Z. (2016). Recessive mutations in TRMT10C cause defects in mitochondrial RNA processing and multiple respiratory chain deficiencies. *Am. J. Hum. Genet.* 99:246. 10.1016/j.ajhg.2016.06.013 27392079 PMC5005466

[B23] Mi H. Huang X. Muruganujan A. Tang H. Mills C. Kang D. (2017). PANTHER version 11: expanded annotation data from gene ontology and reactome pathways, and data analysis tool enhancements. *Nucleic Acids Res.* 45 D183–D189. 10.1093/nar/gkw1138 27899595 PMC5210595

[B24] Murayama E. Kissa K. Zapata A. Mordelet E. Briolat V. Lin H. F. (2006). Tracing hematopoietic precursor migration to successive hematopoietic organs during zebrafish development. *Immunity* 25 963–975. 10.1016/j.immuni.2006.10.015 17157041

[B25] Otten A. B. Smeets H. J. (2015). Evolutionary defined role of the mitochondrial DNA in fertility, disease and ageing. *Hum. Reprod. Update* 21 671–689. 10.1093/humupd/dmv024 25976758

[B26] Otten A. B. C. Kamps R. Lindsey P. Gerards M. Pendeville-Samain M. Muller M. (2019). Tfam knockdown results in reduction of mtDNA copy number, OXPHOS deficiency and abnormalities in Zebrafish embryos. *bioRxiv* [preprint]. 10.1101/843318 PMC7303330 32596237

[B27] Otten A. B. Theunissen T. E. Derhaag J. G. Lambrichs E. H. Boesten I. B. Winandy M. (2016). Differences in strength and timing of the mtDNA bottleneck between zebrafish germline and non-germline cells. *Cell Rep.* 16 622–630. 10.1016/j.celrep.2016.06.023 27373161

[B28] Parisi M. A. Xu B. Clayton D. A. (1993). A human mitochondrial transcriptional activator can functionally replace a yeast mitochondrial HMG-box protein both *in vivo* and *in vitro*. *Mol. Cell. Biol.* 13 1951–1961. 10.1128/mcb.13.3.1951 8441424 PMC359509

[B29] Pauli A. Montague T. G. Lennox K. A. Behlke M. A. Schier A. F. (2015). Antisense oligonucleotide-mediated transcript knockdown in zebrafish. *PLoS One* 10:e0139504. 10.1371/journal.pone.0139504 26436892 PMC4593562

[B30] Powell C. A. Nicholls T. J. Minczuk M. (2015). Nuclear-encoded factors involved in post-transcriptional processing and modification of mitochondrial tRNAs in human disease. *Front. Genet.* 6:79. 10.3389/fgene.2015.00079 25806043 PMC4354410

[B31] Richardson R. Tracey-White D. Webster A. Moosajee M. (2017). The zebrafish eye-a paradigm for investigating human ocular genetics. *Eye* 31 68–86. 10.1038/eye.2016.198 27612182 PMC5233929

[B32] Santos T. A. El Shourbagy S. St John J. C. (2006). Mitochondrial content reflects oocyte variability and fertilization outcome. *Fertil. Steril.* 85 584–591. 10.1016/j.fertnstert.2005.09.017 16500323

[B33] Semino C. E. Allende M. L. (2000). Chitin oligosaccharides as candidate patterning agents in zebrafish embryogenesis. *Int. J. Dev. Biol.* 44 183–193. 10794076

[B34] Shutt T. E. Bestwick M. Shadel G. S. (2011). The core human mitochondrial transcription initiation complex: it only takes two to tango. *Transcription* 2 55–59. 10.4161/trns.2.2.14296 21468229 PMC3062394

[B35] Stackley K. D. Beeson C. C. Rahn J. J. Chan S. S. (2011). Bioenergetic profiling of zebrafish embryonic development. *PLoS One* 6:e25652. 10.1371/journal.pone.0025652 21980518 PMC3183059

[B36] Thomas P. D. Campbell M. J. Kejariwal A. Mi H. Karlak B. Daverman R. (2003). PANTHER: a library of protein families and subfamilies indexed by function. *Genome Res.* 13 2129–2141. 10.1101/gr.772403 12952881 PMC403709

[B37] Torrent M. Chalancon G. de Groot N. S. Wuster A. Madan Babu M. (2018). Cells alter their tRNA abundance to selectively regulate protein synthesis during stress conditions. *Sci. Signal.* 11:eaat6409. 10.1126/scisignal.aat6409 30181241 PMC6130803

[B38] Ventura-Clapier R. Garnier A. Veksler V. Joubert F. (2011). Bioenergetics of the failing heart. *Biochim. Biophys. Acta.* 1813 1360–1372. 10.1016/j.bbamcr.2010.09.006 20869993

[B39] Wagener F. A. Volk H. D. Willis D. Abraham N. G. Soares M. P. Adema G. J. (2003). Different faces of the heme-heme oxygenase system in inflammation. *Pharmacol. Rev.* 55 551–571. 10.1124/pr.55.3.5 12869663

[B40] Wai T. Ao A. Zhang X. Cyr D. Dufort D. Shoubridge E. A. (2010). The role of mitochondrial DNA copy number in mammalian fertility. *Biol. Reprod.* 83 52–62. 10.1095/biolreprod.109.080887 20130269 PMC2888963

[B41] Wan J. Yourshaw M. Mamsa H. Rudnik-Schoneborn S. Menezes M. P. Hong J. E. (2012). Mutations in the RNA exosome component gene EXOSC3 cause pontocerebellar hypoplasia and spinal motor neuron degeneration. *Nat. Genet.* 44 704–708. 10.1038/ng.2254 22544365 PMC3366034

[B42] Yamamoto T. Iwata H. Goto H. Shiratuki S. Tanaka H. Monji Y. (2010). Effect of maternal age on the developmental competence and progression of nuclear maturation in bovine oocytes. *Mol. Reprod. Dev.* 77 595–604. 10.1002/mrd.21188 20575084

[B43] Zhang J. Hamza I. (2018). Zebrafish as a model system to delineate the role of heme and iron metabolism during erythropoiesis. *Mol. Genet. Metab.* 128 204–212. 10.1016/j.ymgme.2018.12.007 30626549 PMC6591114

[B44] Zhang X. Y. Rodaway A. R. (2007). SCL-GFP transgenic zebrafish: *in vivo* imaging of blood and endothelial development and identification of the initial site of definitive hematopoiesis. *Dev. Biol.* 307 179–194. 10.1016/j.ydbio.2007.04.002 17559829

