# Supplementary material for: Tfam Knockdown Results in Reduction of mtDNA Copy Number, OXPHOS Deficiency and Abnormalities in Zebrafish Embryos
Source: Front Cell Dev Biol. 2020 Jun 12;8:381. doi: 10.3389/fcell.2020.00381 (PMC7303330; doi:10.3389/fcell.2020.00381)
Supplement: Supplementary file 1 [file Data_Sheet_1.PDF]

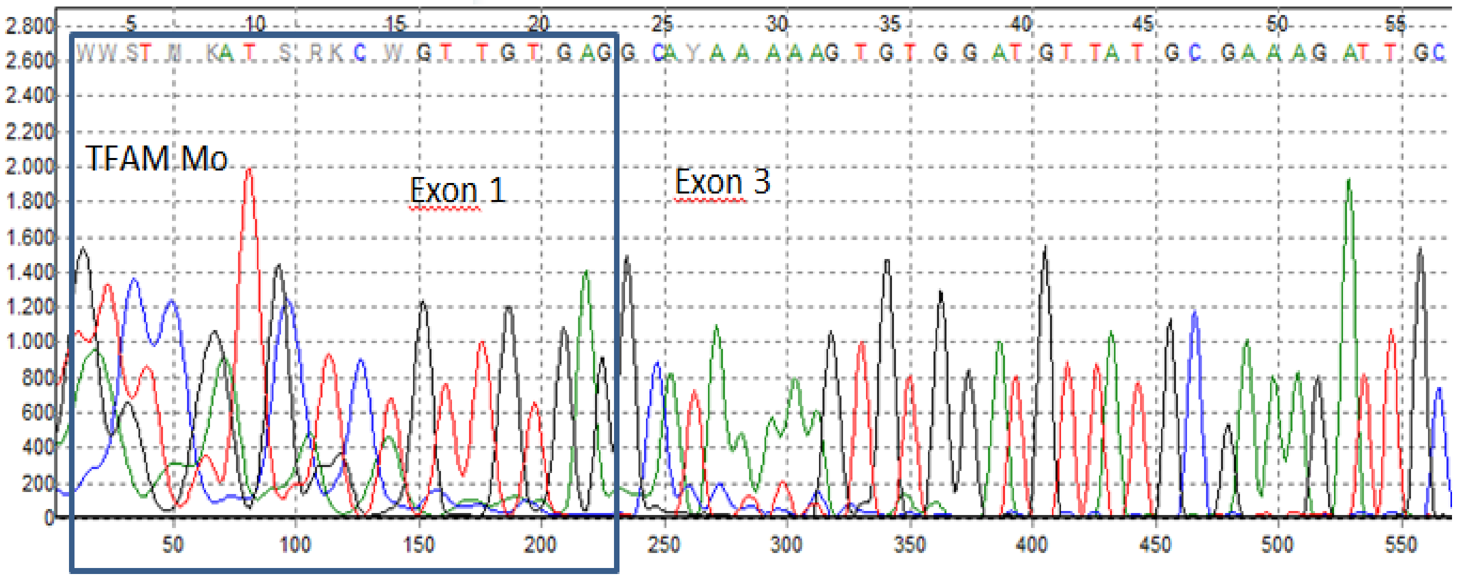


**Supplementary Figure S1.** Sanger sequencing of TFAM after PCR amplification of DNA isolated from *Tfam* splice- MO-treated zebrafish (n= 6 per injected condition from gene expression analysis samples) showed deletion of exon 2 c.84_211del, which predicts a frameshift and premature stopcodon on protein level (p.(Cys29Hisfs*36)).
