# Supplementary material for: Tfam Knockdown Results in Reduction of mtDNA Copy Number, OXPHOS Deficiency and Abnormalities in Zebrafish Embryos
Source: Front Cell Dev Biol. 2020 Jun 12;8:381. doi: 10.3389/fcell.2020.00381 (PMC7303330; doi:10.3389/fcell.2020.00381)
Supplement: Supplementary file 2 [file Data_Sheet_2.pdf]

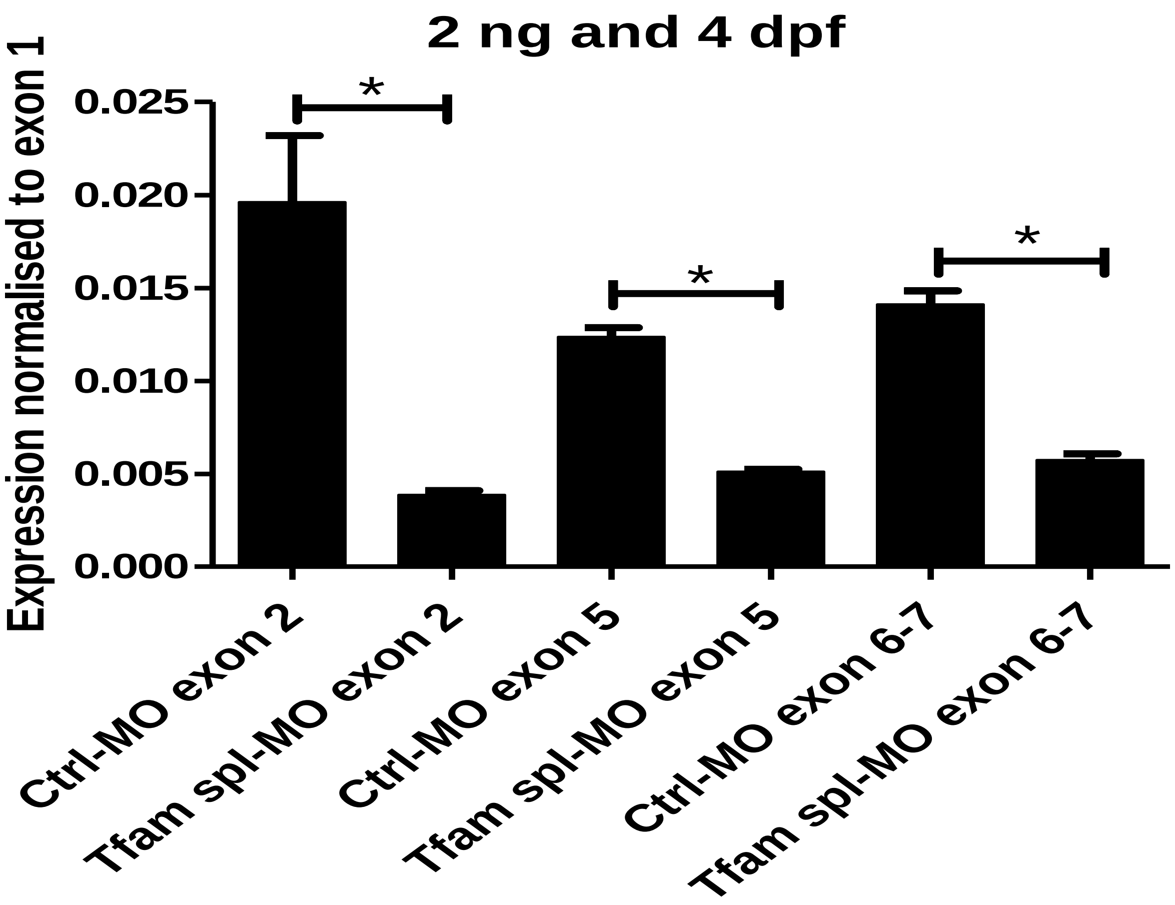

Supplementary Figure S2. Q-PCR analysis of tfam expression. Q-PCR analysis of expression of tfam exon 2,5 and 6-7 at day 4 in zebrafish injected with 2 ng tfam splice-MO compared to the Ctrl-MO (n= 6 per injected condition from gene expression analysis samples). Analysis of tfam exon 5 and exon 6-7 showed respectively 41% and 40% expression in tfam splice-MO injected embryos at day 4. This indicates that ~60% of tfam RNA is subjected to nonsense-mediated decay and that only half of the residual 40% tfam RNA is exon 2 containing wild-type RNA. Bars indicate mean values with SEM. P-values are calculated using ANOVA, * p-value < 0.05.
