# Supplementary material for: Tfam Knockdown Results in Reduction of mtDNA Copy Number, OXPHOS Deficiency and Abnormalities in Zebrafish Embryos
Source: Front Cell Dev Biol. 2020 Jun 12;8:381. doi: 10.3389/fcell.2020.00381 (PMC7303330; doi:10.3389/fcell.2020.00381)
Supplement: Supplementary file 3 [file Data_Sheet_3.PDF]

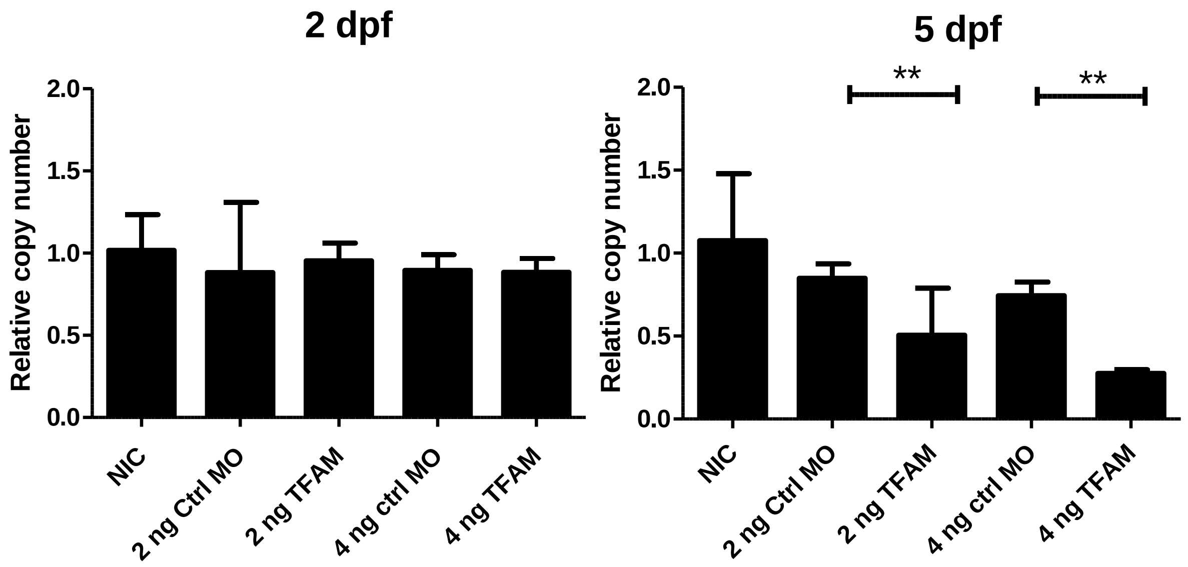

Supplementary Figure S3. mtDNA effect of *Tfam* knockdown on the mitochondrial DNA copy number. The relative mitochondrial DNA copy number has been assessed by mitochondrial ND1 / nuclear B2M ratio at 2 dpf and 5 dpf in non-injected control (NIC, n= 8 per time point) embryos, 2 or 4ng control-morpholino-injected (ctrl-MO) embryos and 2 or 4ng *Tfam* splice-block morpholino injected embryos (n= 20 per injected condition). Data are normalized to the NIC embryos. Bars indicate mean values with SD. *P*-values are calculated using *Bonferroni's Multiple Comparison Test.* * *P*-value < 0.05, ** *P*-value < 0.01.
