# Supplementary material for: Tfam Knockdown Results in Reduction of mtDNA Copy Number, OXPHOS Deficiency and Abnormalities in Zebrafish Embryos
Source: Front Cell Dev Biol. 2020 Jun 12;8:381. doi: 10.3389/fcell.2020.00381 (PMC7303330; doi:10.3389/fcell.2020.00381)
Supplement: Supplementary file 4 [file Data_Sheet_4.PDF]

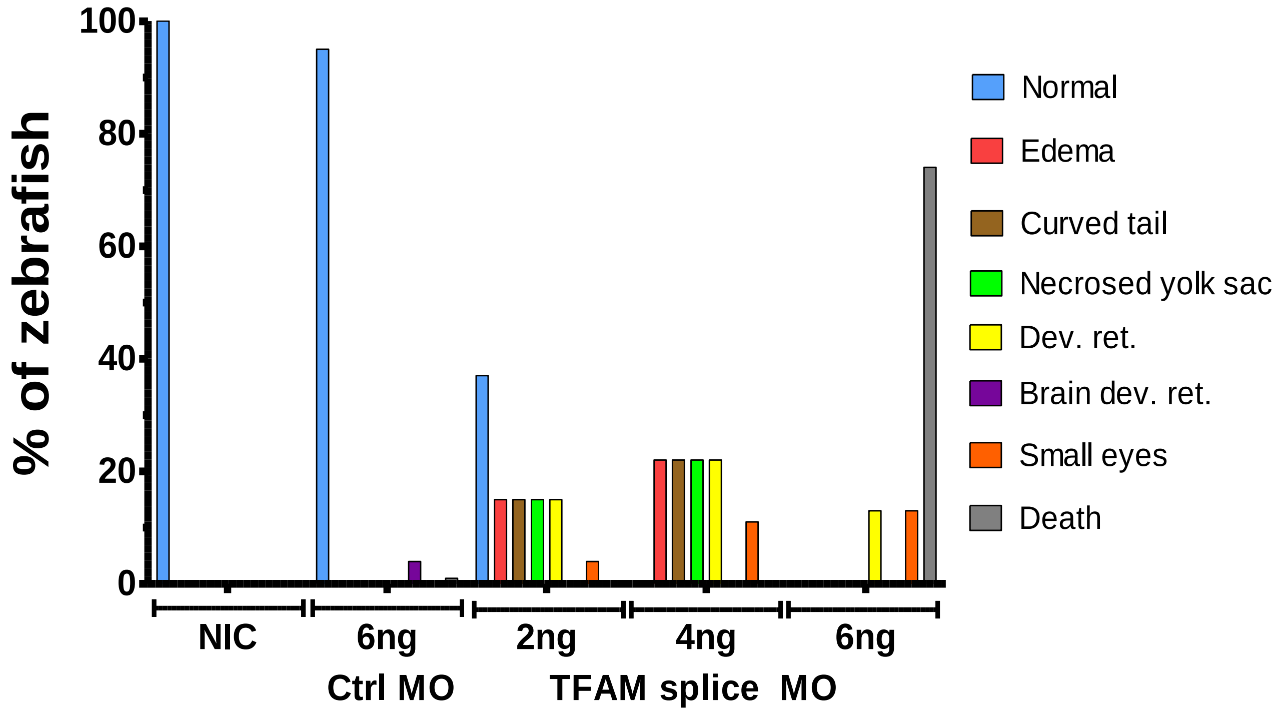


**Supplementary Figure S4.** The macroscopic phenotype (n= 100 per condition) included overall oedema, curved tails, necrotized yolk sacs and small eyes. Fish injected with 4 ng *tfam* splice-MO were more severely affected, as they had a higher count for oedema, curved tails, necrosed yolk sac, small eyes, and developmental delay. The percentage of dead embryos was < 1% for both concentrations of *tfam MO*-injections at 4 dpf.
